# The Effect of the COVID-19 Crisis on Vascular Surgery Training in France

**DOI:** 10.7759/cureus.40863

**Published:** 2023-06-23

**Authors:** Mohammed A Alkhani, Jean Picquet, Xavier Chaufour, Michel Bartoli, Patrick Feugier

**Affiliations:** 1 Vascular Surgery, French College of Vascular and Endovascular Surgery (CFCVE), Paris, FRA; 2 Vascular and Endovascular Surgery Unit, Hôpital Lyon Sud, Lyon, FRA; 3 Vascular and Endovascular Surgery, Claude Bernard University Lyon 1, Lyon, FRA; 4 Vascular and Thoracic Surgery, Centre Hospitalier Universitaire Angers, Angers, FRA; 5 Vascular and Endovascular Surgery, Toulouse University Hospital, Toulouse, FRA; 6 Vascular Surgery, Timone Aortic Center, Timone Hospital, Assistance Publique-Hôpitaux de Marseille, Marseille, FRA

**Keywords:** residency, training, simulation medicine, covid-19, medical education, vascular surgery

## Abstract

In France, since March 2020, the healthcare system has experienced a significant decrease or even suspension of surgical activity and admissions due to the coronavirus disease 2019 (COVID-19).

This activity is essential to the acquisition of technical skills for all trainees enrolled in the Vascular and Endovascular Surgery Training Program either as residents or fellows. The crisis may have affected the training of vascular surgery trainees. We describe the consequences and effects of the COVID-19 crisis on the training of vascular surgery trainees.

A cross-sectional study using an anonymous survey of 12 items was sent to all surgeons in training, registered at the French College of Vascular and Endovascular Surgery (CFCVE). Responses were collected between July and November 2021.

Fifty-two responses were collected from trainees (residents=48%; fellows=52%), seven of who contracted COVID-19 disease. The crisis affected their scheduled and emergency surgical activities, in 96% and 77%, respectively. Thirty-one percent of responders stopped all activity, for an average of 1.5 months.

Eighteen percent of responders were reassigned to other services (emergency department, ICU, vascular access unit, etc...) for an average duration of two months. Sixty-seven percent of responders believe that their level of surgical training was affected due to the crisis.

Fifty-six percent of responders do not think they have achieved their training objectives (55% for fellows, 65% for senior vascular surgery residents (4th, 5th, and 6th year), and 92% for junior vascular surgery residents (year 1, 2, and 3), contributing that to the COVID-19 crisis and its effect on the flow of patients during the crisis. Additional training time (> 3 months) and the utilization of simulation training to reduce the gap produced by the COVID-19 crisis were favored in 60% and 73% of cases respectively.

The COVID-19 health crisis has affected the training of surgical trainees in vascular and endovascular surgery in France. Endovascular and vascular surgical French students in training are waiting now, for additional educational proposals, allowing them to make up for their lack of practice.

## Introduction

Since the declaration of the SARS-Cov-2 (COVID-19) as a pandemic on the 11th of March 2020 by the World Health Organization (WHO), the world has been facing many changes, especially the health care system. In France, to date, there have been more than 33 million cases since the start of the pandemic [1,2]. On the 23rd of March 2020, the French government declared an emergency health state and annulled all elective surgical interventions [3].

The pandemic has not just affected the health care system. It has also affected medical education and residents’ training worldwide, and in France in particular [4-6]. In 2020, the American College of Surgeons (ACS) published its regulating guidelines for vascular surgeons among other guidelines to ensure the best quality of patient care and continuation of care despite the coronavirus disease 2019 (COVID-19) crisis [7]. This unfortunately led to a decrease in access to surgical operations and interventions that are essential to the training of surgical trainees whether residents or fellows.

Several reports have been published in other countries, describing the effect of the COVID-19 crisis, on different surgical specialties such as general surgery [8,9], urology [10], plastic surgery [11], and vascular surgery [12].

We aim to study the effect of the COVID-19 crisis on vascular surgery training in France through a national survey sent to vascular and endovascular surgery residents registered at the French College of Vascular and Endovascular Surgery (CFCVE).

This article was previously presented as a meeting abstract at the thirty-fifth Annual Meeting of the French Language Society of Vascular Surgery, 19-21 May 2022, Marseille, France.

## Materials and methods

An anonymous survey of 12 items was sent to each vascular surgery trainee (residents and fellows) who was registered at the CFCVE, in 2021. There were no exclusion criteria as the survey was meant to assess the effect of the crisis on all vascular surgery trainees in France. The survey was created and distributed by Google Forms. Survey questions were divided into four categories including demographics, surgical formation, surgical activity, and their perspectives on proposed solutions (Table 1). The survey was sent electronically using the CFCVE website, starting in June 2021. Response time to the questionnaire was less than 10 minutes. Questions were either direct multiple choices questions or short-answer questions.

**Table 1:** Survey Questions and Answer Options

	Questions	Responses		
1	Are you a resident or a fellow?	Resident		Fellow
2	What is your level of training?	Resident (1-6)		Fellow (1-2)
3	Do you think that the COVID-19 crisis affected the flow of patients in vascular surgery?	Yes	No	Maybe
4	Do you think that the COVID-19 crisis affected the number of emergency cases?	Yes	No	Maybe
5	Do you think that the crisis has affected your level of surgical training?	Yes	No	Maybe
6	Have you been diagnosed with COVID-19?	Yes	No	
7	Have you been reassigned to other units?	Yes	No	If yes, Where? (ICU, ED, VAU)
8	Have you been out of surgical work during the pandemic? If yes: how long? (months)	Yes	No	
9	After two years of the COVID-19 crisis, did you reach your personal goal of your surgical training?	Yes	No	
10	Did your institute or university suggest the use of simulation labs to reduce the gap produced during the COVID-19 crisis?	Yes	No	
11	Do you think that extra work like simulation training or skills labs can reduce the gap produced during the COVID-19 crisis?	Yes	No	
12	Do you think that adding additional training time can help reduce the gap produced during the COVID-19 crisis?	Yes	No	if yes, for how many months? (1-3/3-6/More)

Senior surgical residents were defined as 4th, 5th, and 6th year of residency, while junior residents were defined as 1st, 2nd, and 3rd years.

The COVID-19 crisis period was defined as the period extending from March 2020 to June 2021. This period was spread over three semesters of university surgical training.

Results were collected between July and November 2021. Three electronic reminders were sent to residents, during this period, as well as to the Union of Residents of our surgical discipline (Société des Internes et Chefs de Clinique de Chirurgie Vasculaire - SICCV). Only complete survey responses were considered. Results are presented as percent response. No consent was needed, as the survey and the collection process were anonymous. The study was approved by the Institutional Review Board of the CFCVE. 

## Results

Two hundred and twenty-six junior members, registered with the CFCVE, were contacted through e-mail. We have collected 52 complete responses (response rate = 23%). Out of the 52 responders, 25 were residents (48%), and 27 were fellows (52%) (Table 2).

**Table 2:** Description of Survey Participants

Residents’ Year of Training (n=30)	Number (%)
1	1(2%)
2	8(15%)
3	7(14%)
4	7(14%)
5	4(7%)
6	3(5%)
Fellows’ Year of Training (n=22)	
1	7(14%)
2	15(29%)

Thirty-one percent of responders (n= 16) reported that they were out of surgical work with an average duration of 1.5 months. Several residents (21%) were deployed to cover and help other units such as the intensive care unit (ICU), the emergency department, and the vascular access unit (VAU). Seven of the trainees (13.5%) were diagnosed with COVID-19 during the pandemic requiring taking time off from their respective training. 

Most responders believe that the pandemic has affected both elective and emergency cases (96% and 77%, respectively). Seventy-seven percent of overall responders believe that the crisis has affected their level of surgical training, contributing to various reasons such as decrement in emergency cases, effect on the flow of patients during the crisis, diagnosis of COVID-19, and reassignment to different other specialties. The majority (n= 29, 56%) believe that they have not reached their personal goal in training. This was the case in 92% and 65% of junior and senior surgical residents, respectively. 

In terms of reducing the training gap caused by the pandemic, 73% of responders believe that the use of simulation is important and can have a positive impact on their practical and technical training for the future. Sixty percent of responders believe that adding extra time of surgical training time (average of 3-6 months) would decrease the gap produced by the COVID-19 crisis. However, only 23% reported that their academic centers had offered or suggested the use of simulation centers. 

## Discussion

Medical education and surgical training have suffered an effect due to the COVID-19 crisis. This effect has had an impact on training and education in various specialties in various domains whether surgical [7-11], medical [13], or even diagnostic medicine [14]. Our study complies with other studies describing the effect on vascular surgery training, where Ilonzo et al. described the effect of the crisis on vascular surgery training in the United States [12]. One main reason for this effect on surgical specialties is that surgical practice was much lower during COVID-19, and this practice is much needed for the formation of surgical trainees.

Our results show similar results to other studies describing the effect of the COVID-19 crisis on the training of residents. Ammann et al. mentioned the decrement in the cases logged by surgical residents. This was also evident with orthopedic surgery fellows where 14% fewer logged arthroplasty procedures during the 2019-2020 academic year when compared to previous years [15,16].

Surgical training consisted of a residency program lasting five years, which continued with a fellowship or assistantship which could be extended for four years. This training or "old reform" made it possible to take the examination for the Complementary Specialized Study Diploma (DESC). This qualification came in addition to the Diploma of Specialized Studies obtained in general surgery.

In 2017, a new reform of the third cycle of medical studies was implemented in France. This reform, still in progress, introduces many changes in the curriculum and training of surgical students. It leads to the disappearance of training in general surgery. Students choose their surgical specialty at the end of the Examen Classant National (ECN). They are entitled, however, to remorse until the second semester of phase 2 or deepening, allowing him to change specialty once.

The training course is divided into three sections: phase 1 or base (two semesters), then phase 2 or deepening (six semesters), and finally phase 3 or consolidation (two years). Before starting the third phase, the student is required to pass their exercise thesis. During the consolidation phase, the trainee is entitled to a "Doctor Junior “position.

The period considered as the "Covid crisis" for the study stretches between March 2020 and June 2021. This period, therefore, affected three semesters of training: the two semesters running from November to May and the semester from May to November 2020.

Three surgical batches of residency were therefore directly impacted by the stopping or slowing down of operating activity. The duration of the Covid crisis period represents 25%, 37%, and 75% of the training durations respectively of the entire Specialized Study Diploma (DES), of the basic and deepening training, and of the final consolidation training. It should be noted that during the Covid crisis period, no vascular surgery resident in France was in phase 3 or the consolidation phase. The fellows who responded to our survey were from the "old reform".

Surgical training is based on the acquisition of scientific knowledge on several specific pathologies and the acquisition of technical skills. These relate to techniques in open surgery, as well as techniques in endovascular surgery. These two types of technical skills are although complementary, very different. The assimilation of basic medical knowledge and evidence was ensured during this period, by the regular analysis of the literature, by teaching sessions in video conferences, and by the sharing of numerous courses put online. On the other hand, the acquisition of technical skills that are usually acquired in daily companionship, exercise in the operating room, experience, and practice was missed during the crisis period.

During the COVID crisis period, practice in the operating room has greatly decreased. As a result, practical training and the management of clinical cases have been reduced, even though it is the surgeon's core business and primary motivation. This practice is important during the last years of training, during which accountability and autonomy in the operating room become essential. This largely explains the responses of the most advanced students in their training, most of whom report not having achieved their training objectives.

Several solutions have been proposed to decrease the gap produced by the pandemic, such as the use of online resources and lectures, the use of simulation labs, and independent learning [17,18]. Although hands-on training is important and essential in surgical training, the use of simulation labs can be effective. Unfortunately, in our study, we found out that the use of simulation was not used extensively. Only 23% of academic institutions or hospitals seem to suggest the use of simulation labs. However, more than 73% of responders believed that simulation can be beneficial. 

Another solution that can be proposed is adding additional training time. Additional time can compensate for the time lost during the pandemic. Surprisingly, most of the responders believe that adding additional training time can be of value in decreasing the time lost during the pandemic. 

Our study has many limitations, and being a survey-based study is one of them. Unfortunately, we also had a low response rate (23%), despite the reminders sent to trainees. Due to the low response rate, only descriptive analysis was made rather than prescriptive. However, it does give an insight into the effect of the pandemic on vascular surgery training.

## Conclusions

It is evident that vascular surgery trainees have suffered an impact on their training due to the pandemic. Although our study focused on vascular surgery trainees, we believe though that this study can show a glimpse of the effect of the pandemic on surgical training in general too. However, the late consequence of this impact is still unknown. Although hands-on experience is essential for the training of surgical trainees, we believe that training in simulation labs and self-learning can however decrease the gap that is produced by the pandemic.
